# Novel liver fibrosis model in *Macaca fascicularis* induced by thioacetamide

**DOI:** 10.1038/s41598-020-58739-4

**Published:** 2020-02-12

**Authors:** Megumi Matsuo, Soichiro Murata, Shunsuke Hasegawa, Yumi Hatada, Masayuki Ohtsuka, Hideki Taniguchi

**Affiliations:** 1 Department of Regenerative Medicine, Yokohama City University Graduate School of Medicine, 3-9, Fuku-ura, Kanazawa-ku, Yokohama, Kanagawa 236-0004 Japan; 2 Department of General surgery, Graduate School of Medicine, Chiba University, 1-8-1, Inohana, Chuo-ku, Chiba, 260-8670 Japan; 3 Division of Regenerative Medicine, University of Tokyo, 4-6-1, Shirokanedai, Minato-ku, Tokyo, 108-8639 Japan

**Keywords:** Hepatology, Inflammation

## Abstract

Although transplantation is the only definitive treatment for liver cirrhosis, there remains a shortage of donors, necessitating that novel treatments be developed. We aimed to establish a liver fibrosis model in *Macaca fascicularis* that can help accelerate preclinical research. Liver fibrosis was induced by administering thioacetamide (TAA) and carbon tetrachloride (CCl_4_). Analysis of residual liver function and fibrosis progression was based on clinical indices, such as the Child–Pugh score or fibrotic markers, besides histology. TAA-induced marked fibrosis, whereas CCl_4_ did not induce fibrosis. Concerning residual liver function, both of TAA and CCl_4_ worsened the indices of the Child–Pugh score, but only the TAA model increased the retention ratio of indocyanine green. The TAA-induced fibrosis model in *Macaca fascicularis* worsens fibrosis and residual liver function, mimicking Child–Pugh grade B. Given that our model was evaluated by clinical indices, it could be applicable to preclinical research.

## Introduction

Liver cirrhosis represents end-stage liver injury in patients with alcohol abuse and chronic hepatitis, ultimately resulting in hepatocellular carcinoma, and is associated with high morbidity and mortality^1,2^. Cirrhosis is characterised by a widespread disruption of the normal liver architecture due to fibrosis and decreased residual liver function, with liver transplantation being the only definitive treatment for patients with end-stage liver injury. However, donor shortages results in high mortality rates among patients in waiting lists^3^, suggesting there is an urgent need to develop alternate approaches.

A major barrier for the development of alternative treatments for end-stage liver injury is the lack of a reliable model of liver fibrosis in non-human primates (NHPs) that can facilitate preclinical research. Although many fibrosis models have been reported in rodents^4,5^, it is unclear how accurately rodent data can predict outcomes in humans because of the various biological and anatomical differences^6^. In contrast, old-world monkeys, such as *Macaca fascicularis* and rhesus monkeys have many anatomical and immunological similarities to humans^6,7^. Therefore, experiments in NHPs have been widely employed when developing vaccines or novel procedures for clinical application^8,9^. To date, however, there have only been few reports of liver fibrosis models in NHPs^10–12^. These existing reports have also failed to cover all relevant clinical indices, such as the Child–Pugh score^13^, Liver damage grade, which was defined by the Liver Cancer Study Group of Japan^14^, and Model for End-Stage Liver Disease (MELD) score^13,15^. These indices are necessary for evaluating residual liver function or prognosis when determining the clinical indication for liver transplantation.

An article previously reported that administering carbon tetrachloride (CCl_4_) with a high fat diet and alcohol for 16 weeks induced liver fibrosis in *Macaca fascicularis*^10^. Fibrotic progression was shown both histologically and serologically by the presence of hyaluronic acid (HA), collage type IV (Col-IV), and type III procollagen-N-peptide (P-III-P). However, the report lacked data about total bilirubin levels or ascites, which are both included in the Child–Pugh score. More recently, a liver fibrosis model was reported in another species of new world monkey, common marmosets, that was induced by thioacetamide (TAA)^12^. They reported four protocols with the appearance of decreasing serum albumin levels and increasing fibrosis in some monkeys. However, they also failed to cover all clinical indices, and the total bilirubin level increased transiently, which deviates from the clinical features of chronic liver diseases. To the best of our knowledge, there are no reports of liver fibrosis models in old-world monkeys induced by TAA.

In this study, we evaluate liver fibrosis models induced by CCl_4_ and TAA in *Macaca fascicularis*, using clinical indices of both residual liver function and fibrotic progression to establish a model that mimicked Child–Pugh grade B to C.

## Results

### General appearance and macroscopic appearance

**Figure 1:**
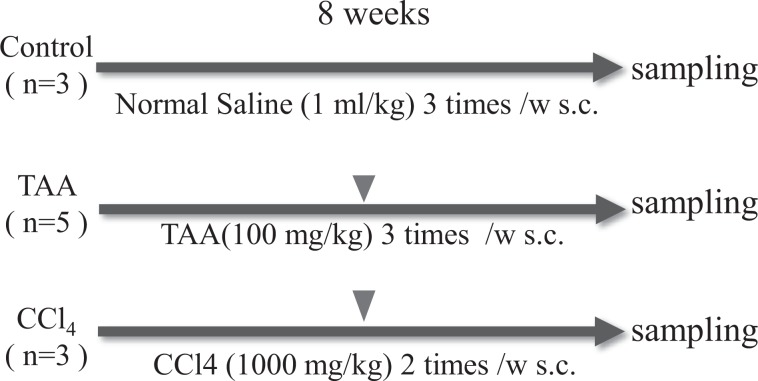
Study protocol.Eleven monkeys were allocated to three groups and received either control, thioacetamide (TAA), or carbon tetrachloride (CCl_4_) for 8 weeks. Inverted triangle = liver biopsy at week 4.

Figure 1 outlines the study protocol and shows the group allocation. Table 1 summarises the general information regarding the allocated monkeys in this study. Monkeys treated by TAA lost appetite and some vomited just after administration during the first week, but their appetites and activities recovered after 2–3 weeks of administration. By contrast, monkeys treated with CCl_4_ did not show any change during the first week and lost their activity and appetite gradually over 3–4 weeks after the initial administration. In addition, subcutaneous administration of CCl_4_ caused skin erosion.

**Table 1:** Summary of general information of each monkey.

	Sex	Age	Initial BW (kg)	Last BW (kg)	Survival (day)	Adverse effects
control-1	F	8	2.74	2.72	56	none
control-2	F	8	2.73	2.65	56	none
control-3	F	12	3.38	3.42	56	none
TAA-1	F	7	3.26	3.04	56	vomiting after administration
TAA-2	F	18	3.17	2.75	5	vomiting after administration
TAA-3	M	6	3.94	2.90	42	vomiting after administration
TAA-4	F	17	3.45	3.03	56	vomiting after administration
TAA-5	M	8	5.00	3.40	56	none
CCl_4_-1	M	10	3.46	3.47	51	skin erosion
CCl_4_-2	F	13	2.98	2.76	49	skin erosion
CCl_4_-3	M	12	5.53	4.08	42	none

Eleven healthy *Macaca fascicularis* (6–17 years old, 3.38 ± 0.86 kg) were allocated into control, TAA treatment and CCl_4_ treatment groups. Mean ± SD.

**Figure 2:**
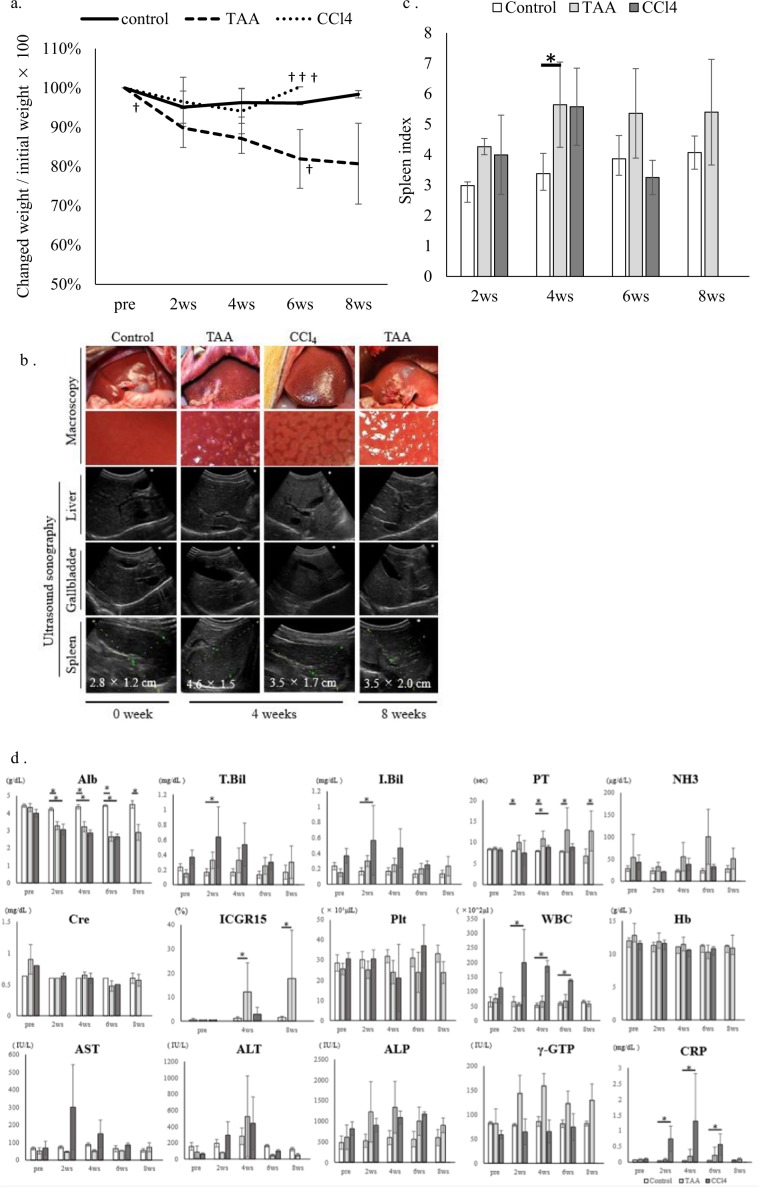
Changes in body weight, ultrasound sonography findings, spleen index and Change in blood chemistry in each group (**a**) Body weight changes per group (mean ± SD): straight line, control group; dashed line, thioacetamide (TAA) treatment; and dotted line, carbon tetrachloride (CCl_4_) treatment. (**b**) Macroscopic and ultrasound changes. Macroscopically, there were changes to the liver surfaces by both reagents. Ultrasound sonography (US) did not show ascites in any group, but did show splenomegaly in the monkeys receiving TAA and CCl_4_ (**c**) Spleen index was calculated by long diameter (cm) × short diameter (cm) of spleen on US. The index significantly increased in monkeys receiving TAA (**d**) Blood chemistry was analysed every 2 weeks except for ICGR15, which was analysed every 4 weeks: white bar, control; light grey bar, TAA treatment; grey bar, CCl_4_ treatment. *p < *0.05* vs control by Mann–Whitney *U* test. Mean ± SD.

Monkeys treated by TAA lost body weight over time (Fig. 2a). Macroscopically, monkeys treated by TAA showed coarse granulation formation on their liver surfaces while those treated by CCl_4_ showed fatty injury like change (Fig. 2b). Abdominal ultrasound revealed that ascites did not develop in any monkeys and any changes in the biliary tract, indicating biliary obstruction or cholecystitis, did not appear in any monkeys (Fig. 2b). On the other hand, splenomegaly appeared in monkeys receiving TAA and CCl_4_ from 2 weeks after initial administration (Fig. 2c).

### Residual liver function

Albumin levels decreased over time in monkeys treated with both TAA and CCl_4_ from 2 weeks after initial administration. The prothrombin time increased persistently in monkeys receiving TAA, whereas the total bilirubin level increased significantly in monkeys receiving CCl_4_. Only TAA increased ICGR15. Serum NH_3_ levels increased in both the TAA and CCl_4_ groups, while aspartate transaminase (AST) and alanine aminotransferase (ALT) increased transiently 2–4 weeks after initial administration and recovered over time. Another feature of chronic liver disease, decreasing platelet levels, was also induced by both TAA and CCl_4_. However, neither model affected creatinine levels. Interestingly, white blood cell and C-reactive protein levels significantly increased in monkeys receiving CCl_4_ (Fig. 2d).

### Histological analysis and progression of liver fibrosis

**Figure 3:**
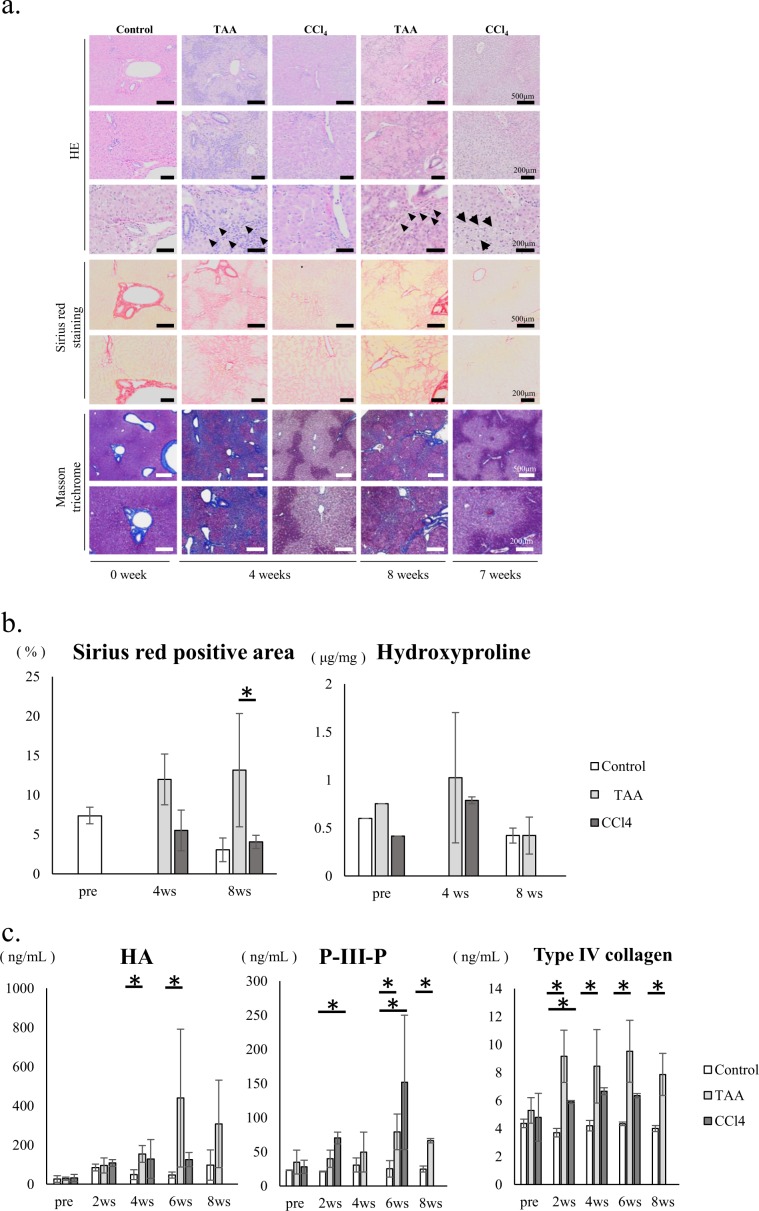
Histological analysis and progression of liver fibrosis. (**a**) Hematoxylin/eosin, Sirius red staining, and Masson trichrome staining: arrow heads in specimens treated by thioacetamide (TAA) show inflammatory infiltration by lymphocytes around the sinusoids at weeks 4 and 8, whereas the arrows in specimens treated by carbon tetrachloride (CCl_4_) show hepatocyte ballooning at week 7. (**b**) Quantitative analysis of the Sirius red positive area by ImageJ and hydroxyproline assay. (**c**) Enzyme-linked immunosorbent assay of hyaluronic acid (HA), collage type IV (Col-IV), and type III procollagen-N-peptide (P-III-P): White bars = control group; light grey bars = TAA treatment; and grey bars = CCl_4_ treatment. *p < *0.05* vs control by Mann–Whitney *U* test. Mean ± SD. Eleven healthy *Macaca fascicularis* (6–17 years old, 3.38 ± 0.86 kg) were allocated into control, TAA treatment and CCl_4_ treatment group. Mean ± SD.

Histology in control monkeys revealed normal cellular architecture (Fig. 3a). Fibrosis developed significantly in monkeys receiving TAA, but was less marked in monkeys receiving CCl_4_ based on the analyses of tissue samples and fibrotic markers. Histology of the biopsy specimens from monkeys receiving TAA at 4 and 8 weeks showed bridging fibrosis, a typical feature of F3 fibrosis, and massive inflammatory infiltration of lymphocytes around sinusoids (arrowhead of Fig. 3a). The histology of monkeys receiving CCl_4_ showed centrilobular congestion but not fibrosis formation at 4 weeks. We conducted a second liver biopsy at 7 weeks because the general condition of monkeys receiving CCl_4_ became poor. We hypothesised that they would not survive until the eighth week. The histology of the biopsy specimen at 7 weeks showed ballooning that are characteristic of fatty liver injury (arrow, Fig. 3a), but did not show significant fibrosis formation.

Quantitative analysis of fibrosis based on the area positive for Sirius red showed fibrotic progression in monkeys receiving TAA but not in those receiving CCl_4_. The hydroxyproline level tended to increase in monkeys receiving TAA, but this increase was not over time as with the other fibrosis assessments (Fig. 3b). HA levels increased 4 weeks after initial dosing with both TAA and CCl_4_; however, it only increased persistently to week 8 in monkeys receiving TAA. The levels of P-III-P and Col-IV increased 2 weeks after initial administration and persisted throughout the administration of both TAA and CCl_4_. Finally, levels of HA and Col-IV increased markedly in monkeys receiving TAA (Fig. 3c).

## Discussion

Liver cirrhosis is characterised by devastation of residual liver function and replacement of the normal parenchyma by fibrosis. Transplantation is the only definitive, albeit radical, treatment for liver cirrhosis, but donor shortages remain an issue. Several novel treatments have been developed, such as cellular transplantation^16^ and molecular targeted drugs^17,18^; however, none of these treatments have proven to be suitable to replace liver transplantation. Therefore, the development of viable alternates continues to be an unmet public health need. To accelerate the development and clinical application of novel treatments or procedures via effective preclinical research, we must first establish reliable models of liver fibrosis in NHPs (e.g., *Macaca fascicularis*), which are more closely related to humans^6^.

Clinical applicability requires that any usable model of liver fibrosis model in NHPs be formulated based on clinically relevant indices. Otherwise, the models may not truly simulate liver cirrhosis in clinical settings. Quantitative analysis of histological data is not always used clinically, but it can be used to establish liver fibrosis in animal models objectively. To date, no model of fibrosis in NHPs has been established based on clinical indices and quantitative analyses of fibrosis. In this study, based on these relevant clinical indices, we showed that TAA and CCl_4_ induced quantitative fibrosis progression in *Macaca fascicularis*.

Liver damage induced by TAA more closely reflected the clinical features of liver cirrhosis than that induced by CCl_4_ in terms of both residual liver function and fibrotic progression in our protocol. Fibrosis progression was much more significant in monkeys receiving TAA than in monkeys receiving CCl_4_. Although histological analysis by biopsy is the most accurate assessment of fibrosis, it is invasive. Therefore, fibrotic markers such as HA, P-III-P and Col-IV are generally used as supplemental markers of fibrosis progression in clinical settings. In this study, each of these markers predicted fibrogenesis and correlated well with the histological findings. Changes in the fibrotic markers showed that TAA administration for 4 weeks induced liver fibrosis and that administration for an additional 4 weeks worsened that fibrosis. Hydroxyproline assays are widely used as a quantitative analysis of fibrosis in animal models, but we did not show a correlation with either the histological results or the fibrotic markers. Given that fibrogenesis is not homogeneous in the liver, hydroxyproline assays of small specimens may not always reflect overall fibrosis formation.

The blood chemistry results indicate that liver damage began 2 weeks after the reagents were initially administered. We considered that these changes indicated the onset of acute liver damage because AST and ALT transiently increased 2–4 weeks after initial administration and recovered over time. Because of this acute injury, a monkey (TAA-2) died just 5 days after administration. To prevent acute death by TAA administration, we should start with a low dose and then gradually increase the dose to the effective dose. Additional administration 2–4 weeks after initial administration induced chronic liver damage and resulted in persistent exacerbation of the clinical indices Child–Pugh score and Liver damage grade. According to the indices of Child–Pugh score, such as albumin, total bilirubin, NH_3_ and prothrombin time, both reagents induced liver damage that mimicked grade B severity by the eighth week. However, only TAA administration worsened ICG clearance function. The ICG clearance test is widely used clinically for dynamic assessments of the capacity of the liver to metabolise and eliminate drugs and it indicated whether liver damage is present. Our study is the first we know of in the published literature to show ICG clearance data in NHPs.

TAA was superior to CCl_4_ in our study because it both induced liver fibrosis progression and worsened residual liver function. Notably, the protocol took only 8 weeks to establish a fibrosis model, which was the shortest time required among the existing models in NHPs, which took more than 16 weeks at least. This could contribute to the accomplishment of the three R’s principle^19^; Replacement, Reduction and Refinement. Furthermore, research with this model could predict effects in humans with greater accuracy because the results were based on clinical indices and objective data specific to fibrosis.

Our study has several limitations. First, we found individual differences in the effects of the reagents. One monkey that received TAA showed neither significant fibrosis formation nor a reduction in liver function, as was apparent in the other monkeys treated by TAA; it may be that the response to reagents is much less predictable in NHPs than in rodents. Individual modifications to reagent administration may be necessary to establish usable models of fibrosis in all NHPs, as previously reported^12^. Second, we could not assess what changes might have occurred after stopping reagent use. Previous reports have indicated that fibrosis can reverse after stopping the reagents in rodents^20^ and NHPs^11,12^. Therefore, it will be essential to determine the effects of stopping TAA and CCl_4_ in future research. Because we strictly complied with the three R’s principle, we designed this study with minimum required monkeys. Additional studies are necessary to verify our results to establish the preclinical model.

In this study, we showed that TAA administration for 8 weeks in *Macaca fascicularis* induces the clinical changes of chronic liver damage consistent with Child–Pugh grade B and class F3 fibrosis (Inuyama classification or Metavir scoring system). This model could prove to be of great benefit in preclinical research for the development of novel treatments for liver cirrhosis.

## Methods

### Animals

All animal studies were approved and performed according to the Tsukuba Primate Research Center. All experiments were performed under the guidelines of the Tsukuba Primate Research Center as well as under the guiding principles for animal experiments using NHPs set out by the Primate Society of Japan. Tsukuba Primate Research Center provided 11 healthy *Macaca fascicularis* (6–18 years old, 3.38 ± 0.86 kg). All monkeys were caged individually in a ventilated room with a 12-h cycle of artificial light from 7 AM to 7 PM. Temperature and humidity were maintained at 23 °C–27 °C and at 50%–70%, respectively.

### Reagent administration

Figure 1 outlines the study protocol and shows the group allocation. TAA (Sigma-Aldrich, St. Louis, MO, USA) was dissolved in normal saline (Otsuka Pharmaceutical, Tokyo, Japan) and administrated three times a week at a dose of 100 mg/kg (n = 5). CCl_4_ (WAKO) was diluted with corn oil (WAKO) and administrated twice a week at a dose of 1000 mg /kg (n = 3). The control group received normal saline at a dose of 1 ml/kg three times a week (n = 3). All reagents were administrated subcutaneously for 8 weeks.

### Assessment of residual liver function

To assess the Child–Pugh score, MELD score and Liver damage grade, blood tests were conducted to determine the levels of albumin, total bilirubin, NH_3_, and creatinine, indocyanine green (ICG) clearance, and prothrombin time to evaluate residual liver function. The ICG clearance test was determined by the retention of ICG at 15 minutes (ICGR15). In addition, AST and ALT levels were measured as general indices of liver damage. Blood was drawn from femoral veins every 2 weeks. We also performed abdominal ultrasonography (AUS) every 2 weeks to determine the presence of ascites and splenomegaly. Spleen index was calculated with short diameter by long diameter of spleen on AUS.

### Assessment of fibrosis progression

Open liver biopsy was conducted before reagent administration and at 4 and 8 weeks after initial administration under xylazine sedation. Tissue samples were then fixed with 10% formalin, embedded in paraffin and stained with hematoxylin and eosin, Sirius red and Masson trichrome. We evaluated fibrosis progression according to the new Inuyama classification^21^ or Metavir scoring system^22^, as follows: F0, no portal fibrosis; F1, portal fibrosis without septa; F2, portal fibrosis with rare septa: F3, numerous septa or lobular distortion without cirrhosis and F4, cirrhosis. The area stained by Sirius red was determined by ImageJ software (National Institute of Health, Bethesda, MD, USA).

We also conducted a hydroxyproline assay for additional quantitative analysis of fibrosis, as previously described^23^. Briefly, homogenous samples of N_4_HCl from solid material were hydrolysed for 12 hours in N_6_HCl at 97 °C. The hydrolysed hydroxyproline was then converted into pyrrole reaction products with a Chloramine T solution (Sigma-aldrich). After adding Ehrlich’s reagent, absorbance was measured by a spectrophotometer at a wavelength of 550–565 nm Enzyme-linked immunosorbent assay of fibrotic markers, such as HA, Col-IV and P-III-P^24^, was also performed by a commercial laboratory (SRL Tokyo Laboratories, Tokyo Japan).

### Statistical analysis

Statistical data were analysed using JMP 13 (SAS Institute Japan, Tokyo, Japan). Mann–Whitney *U* tests were used to compare parameters. Statistical significance was defined by a *p* value of <0.05.
